# Differential Diagnosis of COVID-19 Pneumonia From Influenza A (H1N1) Pneumonia Using a Model Based on Clinicoradiologic Features

**DOI:** 10.3389/fmed.2021.651556

**Published:** 2021-06-15

**Authors:** Wei-Ya Shi, Shao-Ping Hu, Hao-Ling Zhang, Tie-Fu Liu, Su Zhou, Yu-Hong Tang, Xin-Lei Zhang, Yu-Xin Shi, Zhi-Yong Zhang, Nian Xiong, Fei Shan

**Affiliations:** ^1^Department of Radiology, Shanghai Public Health Clinical Center, Fudan University, Shanghai, China; ^2^Department of Radiology, Wuhan Union Red Cross Hospital, Wuhan, China; ^3^Department of Radiology, Zhongshan Hospital, Fudan University, Shanghai, China; ^4^Department of Scientific Research, Shanghai Public Health Clinical Center, Fudan University, Shanghai, China; ^5^Department of Interventional Radiology, Shanghai Public Health Clinical Center, Fudan University, Shanghai, China; ^6^Department of Research and Development, Winning Health Technology Group Co., Ltd., Shanghai, China

**Keywords:** coronavirus disease 2019, influenza A (H1N1), computed tomography, multivariate analysis, differential diagnosis

## Abstract

**Objectives:** Both coronavirus disease 2019 (COVID-19) pneumonia and influenza A (H1N1) pneumonia are highly contagious diseases. We aimed to characterize initial computed tomography (CT) and clinical features and to develop a model for differentiating COVID-19 pneumonia from H1N1 pneumonia.

**Methods:** In total, we enrolled 291 patients with COVID-19 pneumonia from January 20 to February 13, 2020, and 97 patients with H1N1 pneumonia from May 24, 2009, to January 29, 2010 from two hospitals. Patients were randomly grouped into a primary cohort and a validation cohort using a seven-to-three ratio, and their clinicoradiologic data on admission were compared. The clinicoradiologic features were optimized by the least absolute shrinkage and selection operator (LASSO) logistic regression analysis to generate a model for differential diagnosis. Receiver operating characteristic (ROC) curves were plotted for assessing the performance of the model in the primary and validation cohorts.

**Results:** The COVID-19 pneumonia mainly presented a peripheral distribution pattern (262/291, 90.0%); in contrast, H1N1 pneumonia most commonly presented a peribronchovascular distribution pattern (52/97, 53.6%). In LASSO logistic regression, peripheral distribution patterns, older age, low-grade fever, and slightly elevated aspartate aminotransferase (AST) were associated with COVID-19 pneumonia, whereas, a peribronchovascular distribution pattern, centrilobular nodule or tree-in-bud sign, consolidation, bronchial wall thickening or bronchiectasis, younger age, hyperpyrexia, and a higher level of AST were associated with H1N1 pneumonia. For the primary and validation cohorts, the LASSO model containing above eight clinicoradiologic features yielded an area under curve (AUC) of 0.963 and 0.943, with sensitivity of 89.7 and 86.2%, specificity of 89.7 and 89.7%, and accuracy of 89.7 and 87.1%, respectively.

**Conclusions:** Combination of distribution pattern and category of pulmonary opacity on chest CT with clinical features facilitates the differentiation of COVID-19 pneumonia from H1N1 pneumonia.

## Introduction

Coronavirus disease 2019 (COVID-19), caused by the novel severe acute respiratory syndrome coronavirus 2 (SARS-CoV-2, previously known as 2019-nCoV), has become a global health concern that threaten human life and public health security. As of 3 January, 2021, more than 83 million cases and more than 1.8 million deaths have been reported worldwide according to World Health Organization (WHO) statistics (1). When assessing COVID-19, it is noteworthy that influenza viruses occur in the same season. Influenza A (H1N1) is the most common influenza, and it caused a worldwide pandemic in 2009–2010 with more than 18,449 deaths (2) and has now become an annual seasonal influenza, leading to a large number of hospitalizations and deaths (3, 4). Because of the differences in therapy, prognosis, and protective measure between COVID-19 and H1N1, it is important for clinicians and radiologists to identify these two respiroviral infections.

Both COVID-19 and H1N1 pneumonias share similar clinical manifestations, such as mild to moderate flulike syndromes, and are often cured by symptomatic treatments (5, 6). But a few patients develop severe or even lethal acute respiratory distress syndrome (ARDS), particularly in patients with comorbidities (7–11), and their laboratory exams often display lymphopenia, abnormalities in liver function, and myocardial zymogram (7, 8, 10–14).

Significantly, the typical findings on computed tomography (CT) seem different in both pneumonias: bilateral, multifocal ground-glass opacities (GGOs) with or without consolidations or intralobular lines, in a predominant peripheral distribution usually present in COVID-19 pneumonia (15–17); nodules and bud signs (18, 19), bronchiectasis (18, 20), and pleural effusion (20) are common in H1N1 pneumonia but rare in COVID-19 pneumonia (16). Unlike the predominant peripheral distribution in COVID-19 pneumonia, H1N1 pneumonia presents as a predominant peribronchovascular (18, 21) or peripheral (18, 21) or mixed distribution (18, 19). The differences in CT findings in category and the distribution patterns of pulmonary opacity suggest their significance in terms of a differential diagnosis of both pneumonias.

Although it is easy to identify these typical lesions, CT manifestations of COVID-19 and H1N1 pneumonia are very diverse. To date, knowledge regarding the comprehensive identification of both pneumonias still remains limited and cannot meet the urgent clinical needs. Therefore, this retrospective study aimed to assess initial CT and clinical features of the two diseases and further establish a model based on clinicoradiologic features so as to provide some guidance for their early identification.

## Methods

### Study Population

**Figure 1:**
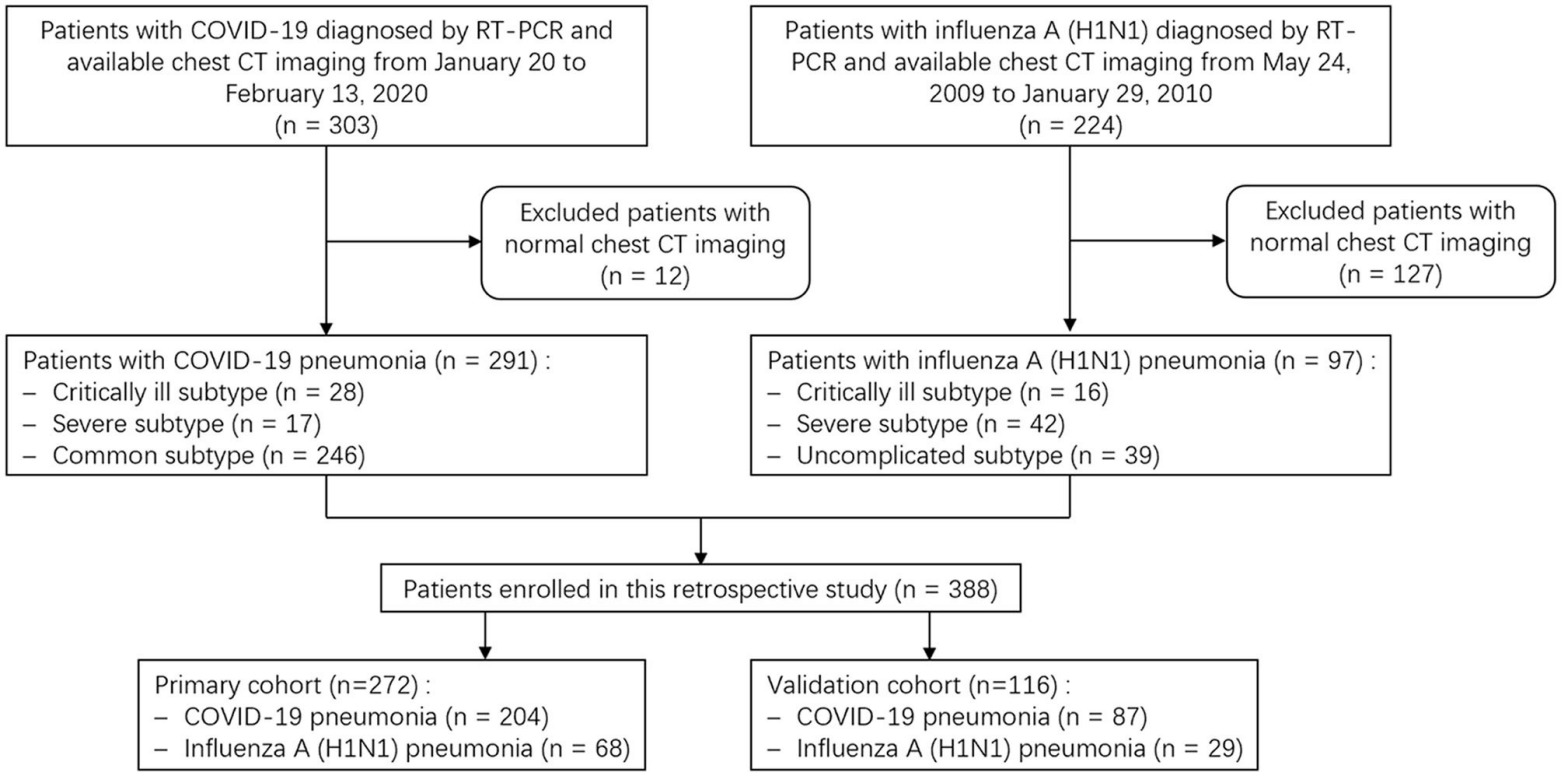
Flow chart shows patient selection, grouping and disease subtypes. COVID-19, coronavirus disease 2019; RT-PCR, real-time Polymerase Chain Reaction.

A search of the medical records in two hospitals' information system was conducted, and 303 COVID-19 patients from January 20 to February 13, 2020, and 224 patients with H1N1 pneumonia from May 24, 2009, to January 29, 2010, were identified. All patients were diagnosed according to the diagnostic criteria of the National Health Commission of China (22, 23) and were confirmed by real-time Polymerase Chain Reaction (RT-PCR) detection of virus nuclear acid. Our exclusion criteria were (a) patients without CT scans; (b) patients with normal CT imaging; and (c) COVID-19 patients with positive influenza A nuclear acid tests. Finally, 291 patients with COVID-19 pneumonia and 97 patients with H1N1 pneumonia were enrolled. All patients were randomly grouped in seven-to-three ratio into a primary cohort (*n* = 272) and a validation cohort (*n* = 116), respectively, by using computer-generated random numbers. The flow chart of patient selection, grouping, and disease subtypes is shown in Figure 1.

### Ethics Statement

This work was approved by the Ethics Committee of Shanghai Public Health Clinical Center, China, and Wuhan Union Red Cross Hospital, China, and informed consent for this retrospective study was waived (YJ-2020-S035-01).

### Clinical Data Collection

The clinical data on admission were retrospectively collected. Particular attention was paid to the demographics, comorbidities, coinfection, symptoms, and laboratory findings. The comorbidities or underlying medical conditions mainly included chronic pulmonary, cardiac, renal and hepatic diseases, diabetes, cerebrovascular disease, hyperlipemia, malignancy, and immunosuppression.

### Imaging Acquiring

All patients underwent CT examinations at full inspiration from the thoracic inlet to the costophrenic angle level. CT scans were performed with one of two scanners (Hitachi Scenaria 64, Hitachi Medical Systems, Tokyo, Japan; or Siemens Sensation 16, Siemens Medical Systems, Forchheim, Germany) using automatic exposure control with the following parameters: tube voltage, 120 or 140 kV; tube current, 150–250 mA; detector width, 64 × 0.625 mm or 16 × 0.75 mm; pitch, 1.57 or 1; rotation time, 0.35 or 0.5 s; field of view (FOV), 350 mm; and matrix, 512 × 512. The reconstruction kernel used was lung smooth with a thickness of 1 mm and an interval of 0.8 mm. The following windows were used: a mediastinal window with a window width of 350 Hounsfield unit (Hu) and a window level of 40 Hu, and a lung window with a width of 1,200 Hu and a level of −600 Hu.

### Image Interpretation

CT images were assessed for the presence and distribution of pulmonary opacities, including pure GGOs, which manifested as a hazy opacity without obscuring the underlying vessels; GGO with interlobular septal thickening or reticulation, which was defined as a crazy-paving sign; GGOs with consolidation, which was defined as an area of opacification obscuring the underlying vessels in GGO; consolidation; centrilobular nodule or tree-in-bud sign, which was regarded present when centrilobular nodules or nodular branching structures resembled a budding tree.

**Figure 2:**
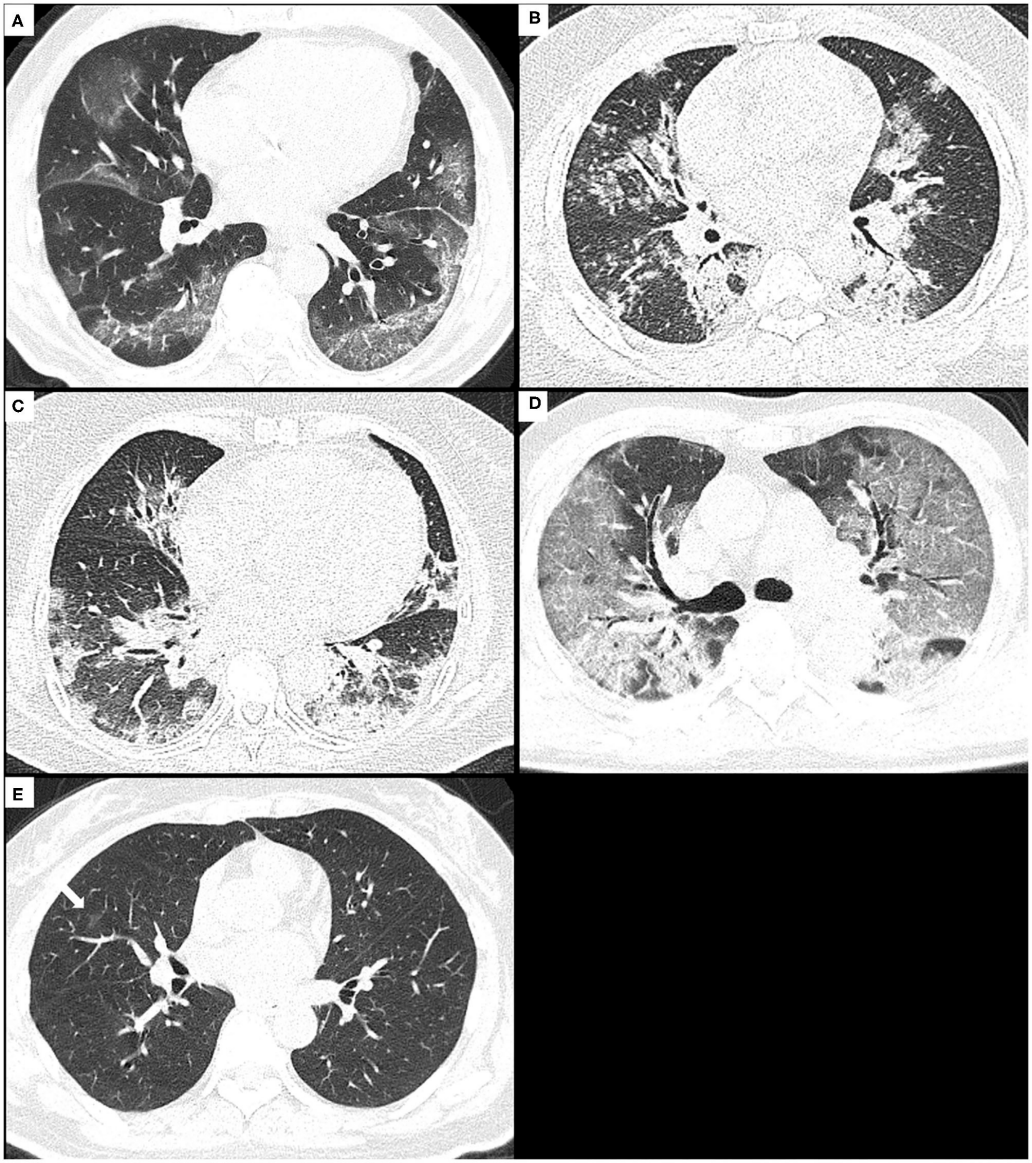
The distribution patterns of pneumonia due to COVID-19 or H1N1. **(A)** A 74-year-old male with COVID-19 pneumonia presents the onset symptom of fever (37.5°C), and the CT shows GGO with consolidation and crazy-paving sign mainly along subpleural lungs, namely, as a peripheral distribution pattern. **(B)** A 29-year-old male with H1N1 pneumonia presents the onset symptoms of fever (39.5°C), cough, and shortness of breath, and the CT shows consolidation and small centrilobular nodules mainly along bronchovascular bundles, namely, as a peribronchovascular distribution pattern. **(C)** A 59-year-old female with H1N1 pneumonia presents the onset symptoms of fever (38.5°C), cough, and shortness of breath, and the CT shows consolidation with GGO along bronchovascular bundles and subpleural lungs, namely, as a distribution pattern of both peripheral and peribronchovascular. **(D)** A 71-year-old male with COVID-19 pneumonia presents the onset symptoms of fever (38°C) and dyspnea, and CT shows diffuse GGO with consolidation and crazy-paving sign in both lungs, namely, as a diffuse distribution pattern. **(E)** A 50-year-old female with COVID-19 pneumonia presents the onset symptoms of fever (39°C) and cough, and CT shows very small non-rounded GGO located in the middle lobe of the right lung lacking a specific distribution. COVID-19, coronavirus disease 2019; GGOs, ground-glass opacities.

The distribution pattern of pulmonary opacities was assessed as being in a predominant peripheral (outer third of the lungs), peribronchovascular, both of peripheral and peribronchovascular, or diffuse distribution, or lacking a specific distribution (Figure 2). The laterality (unilateral and bilateral) and predominant involved pulmonary lobes (upper, middle/lingula, lower, or diffuse) were also assessed.

Bronchial wall thickening or bronchiectasis, focal pulmonary fibrosis (including reticulation and liner opacity), pleural effusion, and mediastinal lymphadenopathy (>1 cm in short-axis diameter) were noted. The number of pulmonary segments involved was counted. All the terms were defined according to the Fleischner Society (24). The images were analyzed independently by two radiologists (Weiya Shi and Fei Shan, with 12 and 19 years of experience in chest radiology, respectively). In cases of disagreement, the results were determined by consensus.

### Statistical Analysis

The continuous data are expressed as the median and interquartile range (IQR, 25th and 75th percentiles), because a majority of them did not follow a normal distribution. The Fisher exact test and the chi-square test were used for categorical variables, and the Wilcoxon rank sum test was used for continuous variables when comparing the clinicoradiologic features of COVID-19 pneumonia with those of H1N1 pneumonia.

In order to evaluate the interobserver agreement (IA) between the two radiologists, the Cohen's Kappa test was used for categorical variables and the intraclass correlation coefficient (ICC) for continuous variables. The kappa coefficient (k) between 0.00 and 0.20; 0.21 and 0.40; 0.41 and 0.60; 0.61 and 0.80; and 0.81 and 1.00 indicated slight, fair, moderate, substantial, and almost perfect agreement, respectively. The ICC values between 0.00 and 0.25; 0.26 and 0.40; 0.41 and 0.60; 0.61 and 0.75; 0.75 and 1.00, indicated poor, low, fair, good, and excellent agreement, respectively.

The clinicoradiologic characteristics found to be significant in univariate analysis were inputted into the least absolute shrinkage and selection operator (LASSO) logistic regression analysis to identify the optimal subset of clinicoradiologic features in order to develop a model for identification. Receiver operating characteristic (ROC) curves were plotted for assessing the performance of the model in the primary and validation cohorts. The cut-off values were defined based on the maximal Youden index.

Statistical analysis was performed with R version 3.6.1 (R Project for Statistical Computing, Vienna, Austria). A two-tailed α < 0.05 was considered statistically significant.

## Results

### Clinical and Laboratory Features

The baseline clinical and laboratory characteristics of the 388 cases were shown in Table 1. Compared with H1N1 pneumonia (97 patients), patients with COVID-19 pneumonia (291 patients) were older (51.0 vs. 31.0 years, *p* < 0.001) and had a lower proportion of men (156/291, 53.6% vs. 66/97, 68.0%, *p* = 0.013). They had lower fever incidence (239/291, 82.1% vs. 93/97, 95.9%, *p* = 0.001) and lower body temperatures (38.0 vs. 38.8°C, *p* < 0.001). H1N1 patients were more likely to have coinfection than COVID-19 patients (16/97, 16.5% vs. 13/291, 4.5%, *p* < 0.001). Among them, all were bacterial infections except for 2 H1N1 cases with bacterial and fungal coinfections.

**Table 1:** Baseline clinical and laboratory characteristics of the patients with COVID-19 pneumonia vs. those with H1N1 pneumonia.

**Characteristics**	**COVID-19 (*n* = 291)**	**H1N1 (*n* = 97)**	***P*-value**
Age, years	51.0 (37.0–64.0)	31.0 (23.0–45.0)	<0.001^*^
**Sex**			
Male	156 (53.6%)	66 (68.0%)	0.013^*^
Female	135 (46.4%)	31 (32.0%)	
Fever	239 (82.1%)	93 (95.9%)	0.001^*^
Body temperature, °C	38.0 (37.5–38.5)	38.8 (38.0–39.5)	<0.001^*^
Onset symptom to hospital admission, d	5.0 (3.0–8.0)	5.0 (2.0–7.0)	0.788
**Comorbidities**	95 (32.6%)	30 (30.9%)	0.754
Chronic pulmonary disease	7 (2.4%)	2 (2.1%)	0.601
Coinfection	13 (4.5%)	16 (16.5%)	<0.001^*^
White blood cell count, × 10^9^/L; normal range: 3.5–9.5	4.7 (3.9–5.9)	4.3 (3.2–6.1)	0.082
Lymphocyte count, × 10^9^/L; normal range: 1.1–3.2	1.1 (0.8–1.5)	1.1 (0.8–1.6)	0.544
Alanine aminotransferase, U/L; normal range: 7–40	22.0 (15.0–33.5)	31.0 (21.0–56.0)	<0.001^*^
AST, U/L; normal range: 13–35	24.0 (19.0–33.0)	41.0 (27.0–65.0)	<0.001^*^
Lactate dehydrogenase, U/L; normal range: 120–250	232.0 (199.0–293.0)	263.5 (191.0–445.0)	0.032^*^
Total bilirubin, μmol/L; normal range: 3.4–20.5	8.1 (6.5–10.5)	9.0 (7.3–12.0)	0.011^*^
Albumin, g/L; normal range: 40.0–55.0	40.8 (37.9–43.1)	40.0 (35.2–45.4)	0.341
Blood urea nitrogen, mmol/L; normal range: 2.6–7.5	4.4 (3.6–5.5)	3.9 (3.1–5.1)	0.017^*^
Serum creatinine, μmol/L; normal range: Male, 53–106; Female, 44–97			
Increased	21 (7.2%)	1 (1.0%)	0.023^*^
C-reactive protein, mg/L; normal range: <3.0	14.8 (5.1–33.0)	22.7 (11.2–51.6)	0.001^*^
CD4^+^ T counts, cell/μL; normal range: 410–1,590	417.0 (291.0–618.0)	365.0 (230.0–535.0)	0.010^*^

*Continuous variables are presented as median (interquartile range), and dichotomous variables are presented as numbers of patients, with percentages in parentheses*.

* *P < 0.05; P-values are from Fisher's exact test or Wilcoxon rank-sum test when comparing characteristics of COVID-19 pneumonia with those of H1N1 pneumonia; COVID-19, coronavirus disease 2019; AST, aspartate aminotransferase*.

The laboratory exams displayed lower serum levels of alanine aminotransferase, aspartate aminotransferase (AST), lactate dehydrogenase, total bilirubin, and c-reactive protein, higher level of CD4^+^ T counts and blood urea nitrogen, as well as more frequency of increased serum creatinine in COVID-19 pneumonia in contrast to those in H1N1 pneumonia (*p* < 0.001, *p* < 0.001, *p* = 0.032, 0.011, 0.001, 0.010, 0.017, and 0.023, respectively).

### CT Characteristics

The IA between the two radiologists was almost perfect for all CT findings except the tree-in-bud sign, for which it was substantial (k = 0.789).

The baseline CT characteristics of the 388 cases were shown in Table 2. In terms of lesions' distribution pattern, 90.0% (262/291) of COVID-19 pneumonia had a peripheral distribution pattern in contrast to only 20.6% (20/97) in H1N1 pneumonia (*p* < 0.001). In the H1N1 pneumonia, a peribronchovascular distribution pattern (52/97, 53.6%) was the most common, though this is rare in COVID-19 pneumonia (9/291, 3.1%, *p* < 0.001). The H1N1 pneumonia was more likely to exist in a distribution pattern of both peripheral and peribronchovascular (17/97, 17.5% vs. 14/291, 4.8%, *p* < 0.001) or be lacking a specific distribution (6/97, 6.2% vs. 3/291, 1.0%, *p* = 0.003). The incidence of diffuse distribution pattern was 1.0% (3/291) and 2.1% (2/97) in COVID-19 pneumonia and H1N1 pneumonia, respectively, and had no significant difference.

**Table 2:** Baseline CT characteristics of the patients with COVID-19 pneumonia vs. those with H1N1 pneumonia.

**Characteristics**	**COVID-19 (*n* = 291)**	**H1N1 (*n* = 97)**	***P*-value**	**Agreement (k or ICC; 95% CI)**
**Main pulmonary opacities**				
pGGO	118 (40.5%)	37 (38.1%)	0.675	0.941 (0.903–0.973)
Crazy-paving sign	206 (70.8%)	39 (40.2%)	<0.001^*^	0.884 (0.835–0.931)
GGO with consolidation	216 (74.2%)	49 (50.5%)	<0.001^*^	0.915 (0.865–0.956)
Consolidation	91 (31.3%)	79 (81.4%)	<0.001^*^	0.912 (0.870–0.948)
Centrilobular nodule or tree-in-bud sign	3 (1.0%)	43 (44.3%)	<0.001^*^	0.789 (0.687–0.891)
NO. of pulmonary segments involved	9 (4–15)	12 (3–17)	0.183	0.998 (0.997–0.998)
**Laterality**				
Bilateral involvement	241 (82.8%)	71 (73.2%)	0.039^*^	1.000 (1.000–1.000)
**Involved pulmonary lobes**				0.948 (0.916–0.972)
Upper	29 (10.0%)	10 (10.3%)	0.922	
Middle/lingula	12 (4.1%)	12 (12.4%)	0.003^*^	
Lower	172 (59.1%)	52 (53.6%)	0.342	
Diffuse	78 (26.8%)	23 (23.7%)	0.548	
**Distribution pattern**				0.877 (0.827–0.925)
Peripheral	262 (90.0%)	20 (20.6%)	<0.001^*^	
Peribronchovascular	9 (3.1%)	52 (53.6%)	<0.001^*^	
Peripheral + Peribronchovascular	14 (4.8%)	17 (17.5%)	<0.001^*^	
Diffuse	3 (1.0%)	2 (2.1%)	0.436	
Lacking a specific distribution	3 (1.0%)	6 (6.2%)	0.003^*^	
**Other signs**				
Bronchial wall thickening or bronchiectasis	18 (6.2%)	28 (28.9%)	<0.001^*^	0.805 (0.692–0.891)
Focal pulmonary fibrosis	103 (35.4%)	21 (21.6%)	0.012^*^	0.928 (0.886–0.965)
Pleural effusion	14 (4.8%)	27 (27.8%)	<0.001^*^	1.000 (1.000–1.000)
Mediastinal lymphadenopathy	4 (1.4%)	1 (1.0%)	0.795	1.000 (1.000–1.000)

*Continuous variables are presented as median (interquartile range), and dichotomous variables are presented as numbers of patients, with percentages in parentheses*.

* *P < 0.05; P-values are from Fisher's exact test or Wilcoxon rank-sum test when comparing characteristics of COVID-19 pneumonia with those of H1N1 pneumonia. For categorical variables and continuous variables, the interobserver agreement is assessed with the kappa coefficient (k) and the intraclass correlation coefficient (ICC), respectively. The k and ICC values are reported with 95% confidence intervals (95% CI). COVID-19, coronavirus disease 2019; GGOs, ground-glass opacities*.

With respect to other CT characteristics, the COVID-19 pneumonia was more likely to present a crazy-paving sign (206/291, 70.8% vs. 39/97, 40.2%, *p* < 0.001), GGO with consolidation (216/291, 74.2% vs. 49/97, 50.5%, *p* < 0.001), bilateral involvement (241/291, 82.8% vs. 71/97, 73.2%, *p* = 0.039), and focal pulmonary fibrosis (103/291, 35.4% vs. 21/97, 21.6%, *p* = 0.012); H1N1 pneumonia, however, was more likely to present consolidation (79/97, 81.4% vs. 91/291, 31.3%, *p* < 0.001), centrilobular nodule or tree-in-bud sign (43/97, 44.3% vs. 3/291, 1.0%, *p* < 0.001), predominant middle/lingula involvement (12/97, 12.4% vs. 12/291, 4.1%, *p* = 0.003), bronchial wall thickening or bronchiectasis (28/97, 28.9% vs. 18/291, 6.2%, *p* < 0.001), and pleural effusion (27/97, 27.8% vs. 14/291, 4.8%, *p* < 0.001).

### Performance of the Model for Differential Diagnosis

**Figure 3:**
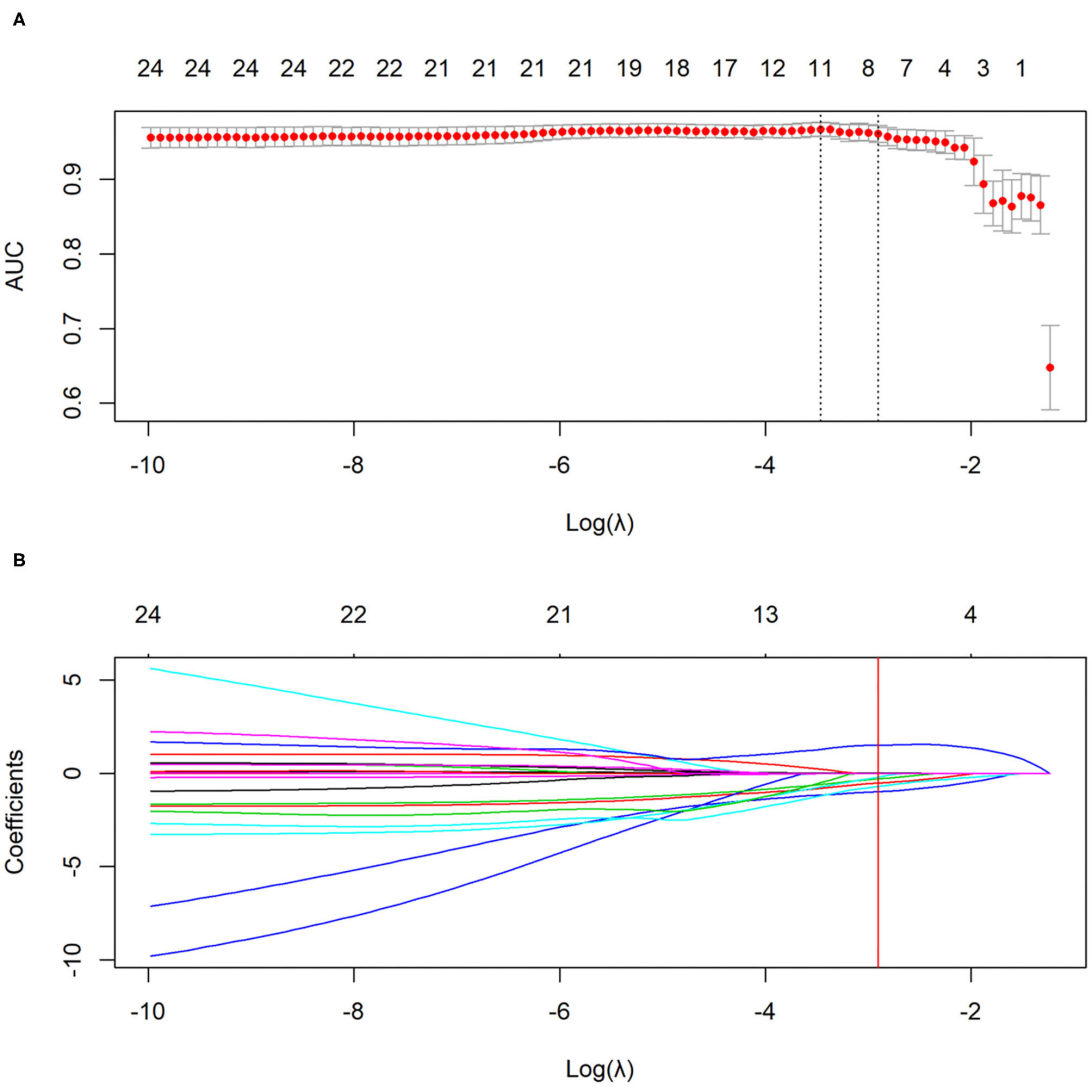
The selection of clinico-radiological features using LASSO logistic regression. **(A)** Optimal feature selection according to AUC value. **(B)** LASSO coefficient profiles of the features. Vertical line is drawn at the selected value using 10-fold cross-validation, where optimal λ results in eight non-zero coefficients. LASSO, least absolute shrinkage and selection operator; AUC, area under curve.

LASSO logistic regression analysis was applied to identify the most valuable clinico-radiological features for differentiating COVID-19 pneumonia from H1N1 pneumonia when the optimal value of log (λ) was −2.906 according to 10-fold cross-validation (Figure 3). The optimal features subset and their coefficient values were shown in Table 3. LASSO-based feature selection revealed that age and peripheral distribution patterns were positively associated with COVID-19 pneumonia, and body temperature, AST, consolidation, centrilobular nodule or tree-in-bud sign, bronchial wall thickening or bronchiectasis, and a peribronchovascular distribution pattern were inversely associated with COVID-19 pneumonia.

**Table 3:** The coefficients of the elected features by LASSO logistic regression analysis.

**Feature selected**	**Coefficient**
Age, years	0.013
Body temperature, °C	−0.523
AST, U/L	−2.998e-03
Consolidation (yes vs. no)	−0.300
Centrilobular nodule or tree-in-bud sign (yes vs. no)	−0.984
Bronchial wall thickening or bronchiectasis (yes vs. no)	−0.207
Peripheral distribution pattern (yes vs. no)	1.505
Peribronchovascular distribution pattern (yes vs. no)	−0.712

*LASSO, least absolute shrinkage and selection operator; AST, aspartate aminotransferase*.

**Figure 4:**
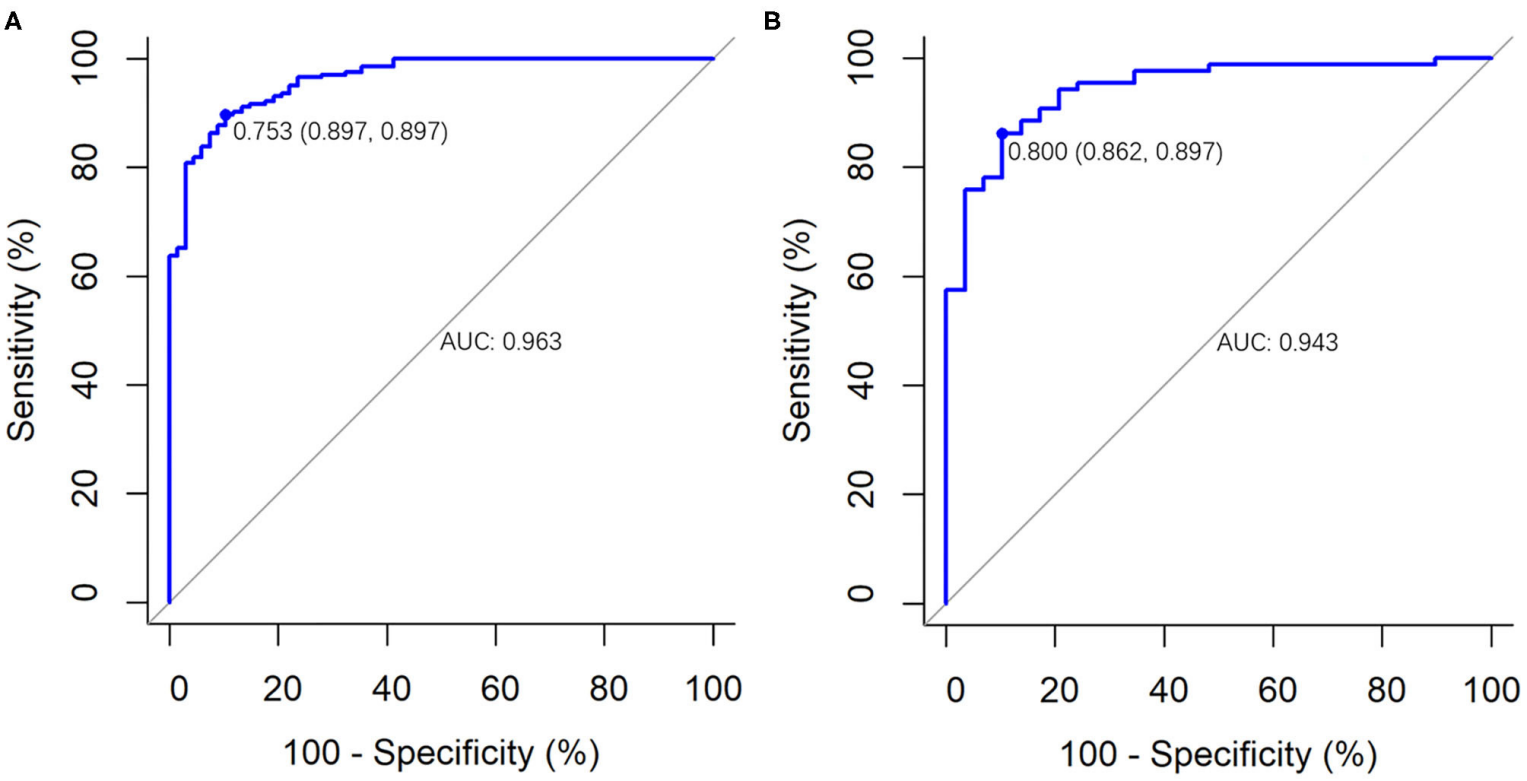
The performance of the LASSO logistic regression model for differentiating COVID-19 pneumonia from influenza A (H1N1) pneumonia. It was presented by ROC curves in **(A)** the primary cohort (AUC, 0.963; 95% CI: 0.942–0.984) and **(B)** the validation cohort (AUC, 0.943; 95% CI: 0.900–0.986).

For the primary and validation cohorts, the LASSO model containing above eight features yielded an area under curve (AUC) of 0.963 (95% CI: 0.942–0.984) and 0.943 (95% CI: 0.900–0.986), with sensitivity of 89.7 and 86.2%, specificity of 89.7 and 89.7%, accuracy of 89.7 and 87.1%, positive predictive value of 96.3 and 96.1%, and negative predictive value of 74.4 and 68.4%, respectively (Table 4 and Figure 4).

**Table 4:** AUC values of the LASSO regression model for differentiating COVID-19 pneumonia from H1N1 pneumonia in the primary and validation cohorts.

**LASSO regression model**	**AUC value (95% CI)**	**Sensitivity (%)**	**Specificity (%)**	**Accuracy (%)**	**PPV (%)**	**NPV (%)**
Primary cohort	0.963 (0.942–0.984)	89.7	89.7	89.7	96.3	74.4
Validation cohort	0.943 (0.900–0.986)	86.2	89.7	87.1	96.1	68.4

*COVID-19, coronavirus disease 2019; AUC, area under curve; LASSO, least absolute shrinkage and selection operator; PPV, positive predictive value; NPV, negative predictive value; 95% CI, 95% confidence intervals*.

## Discussion

RT-PCR detection of viral nuclear acid is widely used for diagnosis and conformation of COVID-19; however, its sensitivity is largely affected by the disease phase, viral loading, and sampling (25). Thus, the routine chest CT is a more sensitive and rapid method (25), and the expert consensus of the Radiological Society of North America (RSNA) has provided guidance to report CT findings attributable to COVID-19 pneumonia (16); however, the reported specificities of CT are low so far, ranging from 25 to 53% (16). Because of the distinct treatments and prognoses between COVID-19 and H1N1, we aimed to accurately identify these two diseases. In our study, CT manifestations of consolidation, centrilobular nodule or tree-in-bud sign, bronchial wall thickening or bronchiectasis, peripheral distribution pattern and peribronchovascular distribution pattern, together with the clinical features such as age, body temperature and AST were identified the optimal features subset for differentiating COVID-19 pneumonia from H1N1 pneumonia. Our model had high diagnostic efficiency (AUC, 0.943; sensitivity, 86.2%; specificity, 89.7%; accuracy, 87.1%) in the validation cohort, which provides guidance for clinical diagnosis.

Several previous studies have found that COVID-19 typically presents GGO with or without consolidation in a peripheral distribution (15–17), which was endorsed by the Society of Thoracic Radiology, the American College of Radiology, and RSNA (16) as guidance for COVID-19 diagnosis. Our study was consistent with previous reports that the COVID-19 pneumonia mainly presented a peripheral distribution pattern (262/291, 90.0%); in contrast, H1N1 pneumonia most commonly presented a peribronchovascular distribution pattern (52/97, 53.6%). The differences in CT imaging between these two pneumonias may result from their distinct pathological changes in lungs. The pathological findings of COVID-19 pneumonia include exudative diffuse alveolar damage with alveolar and interstitial edema, alveolar fibrinous exudate with hyaline membranes, and reactive pneumocytes (26), whereas H1N1 pneumonia, in addition to diffuse alveolar damage, is usually accompanied by necrotizing bronchiolitis and alveolar hemorrhage (27). These pathologic lesion-dependent distribution patterns, which are very conspicuous at the first glance of the images, are valuable indicators for differentiating COVID-19 pneumonia from H1N1 pneumonia.

The bronchiolitis causes central lobular nodules or tree-bud signs, and bronchial wall thickening or bronchiectasis; therefore, it is quite understandable that these two signs were more common in H1N1 pneumonia than in COVID-19 pneumonia (both *p* < 0.001), consisting with other studies (16, 18, 21). We also found that consolidation was more frequent in H1N1 pneumonia (79/97, 81.4%) than in COVID-19 pneumonia (91/291, 31.3%), which is consistent with previous studies and is possibly associated with pathologic basis (8), disease progression or more severe disease (28), bacterial coinfection (29). The latter phenomenon was also found in our study, that is, coinfection (mainly bacterial infection) was more frequent in H1N1 pneumonia (16/97, 16.5%) than in COVID-19 pneumonia (13/291, 4.5%).

Besides radiological features, clinical features such as age, body temperature, and AST should be also taken into consideration for differentiation. Our cohort showed an older median age of the COVID-19 patients; however, it should be viewed cautiously, the statistics were based on the early stage of the outbreak in Shanghai and Hubei province, China. With the global spread, it has been found that the youth are also a susceptible population (30).

Although the model had high diagnostic efficiency, it should be noted that it had a negative predictive value of 68.4% in the validation cohort due to the misdiagnosis of 12 cases of COVID-19 pneumonia as H1N1 pneumonia. The misdiagnosed patients were relatively young (median, 38.5 years; IQR, 32.5–58.5), with high fever (median, 39.0°C; IQR, 38.3–39.7) and moderately elevated AST (median, 30.0 U/L; IQR, 22.5–48.0), and 75.0% (9/12) of them presented consolidation and 58.3% (7/12) a non-peripheral distribution pattern (two cases with a peribronchovascular distribution pattern, four cases with a distribution pattern of both peripheral and peribronchovascular, and one case with a diffuse distribution pattern). Radiologists should pay more attention to these atypical clinicoradiologic manifestations of COVID-19 in young individuals so as to avoid misdiagnosis.

However, our study had limitations. Firstly, there was an imbalance between the sample sizes of COVID-19 pneumonia and H1N1 pneumonia, and the proportion of severe and critically ill H1N1 patients was greater than those of the COVID-19 cohort, which may have led to statistical disequilibrium. Secondly, there is a bias in the laboratory tests because there were several laboratory changes in COVID-19 patients compared to H1N1 patients, using only the tests common to both groups. Thirdly, patients comorbid with chronic pulmonary disease were not excluded, which may lead to a bias in this study, although the proportion of these patients was very low and there was no statistically significant difference between both groups. Fourthly, coinfection is common in patients of both groups, especially in patients with N1H1. Due to the complex nature of the clinical situation, we believe that differential diagnosis is also necessary for these patients to obtain effective subsequent treatment. Therefore, we did not exclude these patients when constructing our analysis model, but this may lead to a bias.

In conclusion, CT characteristics, including the distribution pattern and category of pulmonary opacity, combined with clinical features, can help the early differentiation of COVID-19 pneumonia from H1N1 pneumonia. CT manifestations of peripheral distribution patterns, together with older age, low-grade fever, and slightly elevated AST, indicate COVID-19 pneumonia; however, CT presentations of peribronchovascular distribution patterns, centrilobular nodule or tree-in-bud sign, consolidation, and bronchial wall thickening or bronchiectasis, together with younger age, hyperpyrexia and higher level of AST, suggest H1N1 pneumonia.

## Data Availability Statement

The original contributions presented in the study are included in the article/supplementary material, further inquiries can be directed to the corresponding author/s.

## Ethics Statement

The studies involving human participants were reviewed and approved by the Ethics Committee of Shanghai Public Health Clinical Center, China, and Wuhan Union Red Cross Hospital, China, and informed consent for this retrospective study was waived (YJ-2020-S035-01). Written informed consent from the participants' legal guardian/next of kin was not required to participate in this study in accordance with the national legislation and the institutional requirements.

## Author Contributions

W-YS and FS participated in the study design and conceptualization. W-YS, X-LZ, H-LZ, SZ, and S-PH participated in the acquisition of data. W-YS, FS, and Y-HT participated in analysis and interpretation of data. W-YS and Y-HT participated in drafting of the manuscript and participated in the statistical analysis. FS and T-FL participated in critical revision of the manuscript for important intellectual content. Z-YZ, Y-XS, and NX participated in administrative, technical, material support, and participated in study supervision. All authors contributed to the article and approved the submitted version.

## Conflict of Interest

Y-HT was employed by Winning Health Technology Group Co., Ltd., Shanghai, China. The remaining authors declare that the research was conducted in the absence of any commercial or financial relationships that could be construed as a potential conflict of interest.
